# A Possible Clinical Scenario of Iodine-Containing Supplement Causing Thyroid Dysfunction in Pregnancy

**DOI:** 10.7759/cureus.97741

**Published:** 2025-11-25

**Authors:** Alamin Alkundi, Jane Ohize, Rabiu Momoh

**Affiliations:** 1 Diabetes and Endocrinology, East Kent Hospitals University NHS Foundation Trust, Ashford, GBR; 2 Internal Medicine, William Harvey Hospital, Ashford, GBR; 3 Critical Care, Medway Maritime Hospital, Gillingham, GBR

**Keywords:** case report, iodine, nutritional supplements, patient education, pharmaceutics, pre-conceptual care, pregnancy, public health, thyroid disorders, thyrotoxicosis

## Abstract

This report presents the case of a 33-year-old female patient with a history of Graves’ disease who began using a dietary supplement (containing 150 µg of iodine and other micronutrients) to support conception. There was a laboratory finding of suppressed thyroid-stimulating hormone, high free triiodothyronine, and normal free thyroxine (suggesting thyrotoxicosis) uncovered during an early pregnancy assessment. Her thyroid levels returned to normal after she discontinued the supplement without the need for medical treatment. This case stimulated further review of the literature for the potential risks associated with improper use of iodine-containing supplements without consideration for underlying thyroid conditions and for other possible reasons for this finding.

## Introduction

According to the Dietary Supplement Health and Education Act of 1994, a dietary supplement is any non-tobacco product designed to enhance diet. It must contain one or more dietary ingredients, such as vitamins, minerals, herbs or botanicals, amino acids, or similar substances, and be intended for oral consumption in forms such as pills, capsules, tablets, or liquids. Additionally, the product must clearly state on its front label that it is a dietary supplement [1]. Dietary supplements are commonly taken for a variety of perceived benefits, including strengthening the immune system, improving fertility, promoting hair growth, and enhancing sexual performance. In 2020, usage rates were notable, with approximately 36% of adults in the United Kingdom, 43% in Australia, and 51% in Denmark reported to be using supplements [2].

Our case report suggests that patients with prior Graves’ disease can be particularly susceptible to iodine-containing supplement-induced thyroid dysfunction. The patient under review consumed a multivitamin containing iodine, with a notably disrupted thyroid function on laboratory evaluation at 10-week estimated gestational age. Each tablet provided 150 µg of iodine, the recommended daily intake for adults. As iodine is readily available through various foods, most people typically fulfil their daily needs through diet alone. Existing literature evidence suggests that those with a history of thyroid disease may be more vulnerable to thyroid dysfunction caused by excessive iodine consumption [3].

The thyroid gland is a butterfly-shaped endocrine organ located in the neck that produces thyroxine (T4) and triiodothyronine (T3), hormones essential for regulating energy metabolism, thermogenesis, and protein synthesis. Its activity is controlled by thyroid-stimulating hormone (TSH) from the pituitary. A critical component of thyroid physiology is iodine metabolism: iodine is actively taken up by thyroid follicular cells and incorporated into thyroglobulin to form T3 and T4. Because the human body cannot synthesize iodine, dietary intake is vital, and its deficiency can lead to goiter and hypothyroidism. Adequate iodine ensures proper hormone production, which is especially important for neurological development in fetuses [4].

## Case presentation

A clinically euthyroid female patient in her early 30s, at 10 weeks’ estimated gestational age, was reviewed at an endocrinology clinic. She had a previous history of Graves’ disease (diagnosed 10 years prior, which was managed with oral carbimazole and achieved complete resolution of symptoms clinically and biochemically after four years). She had no other comorbidity. She was referred by her community general practitioner (GP) on account of a new deranged thyroid function test result in pregnancy, showing low serum TSH <0.05 mU/L (reference = 0.5-4 mU/L), an elevated serum free T3 level at 7.4 pmol/L (reference = 3.1-6.8 pmol/l), and a normal serum free T4 at 15 pmol/L (reference = 10-22 pmol/l) (Table 1). Her body mass index was 19 kg/m² (reference = 18-25 kg/m²), and the rest of her physical examination was essentially normal, as were her vital signs on the clinic visit.

**Table 1:** Serial thyroid function test results done on the patient while using the nutritional supplement and post-discontinuation. During supplement use, TSH levels were significantly reduced while FT3 showed a mild increase, findings indicative of thyrotoxicosis. These abnormalities resolved and returned to normal in the periods following the discontinuation of the supplement use. TSH = thyroid-stimulating hormone; T3 = triiodothyronine; T4 = thyroxine

Time point	TSH (reference = 0.5–4.0 mU/L)	Free T4 (reference = 10–22 pmol/L)	Free T3 (reference = 3.1–6.8 pmol/L)
During supplement use	<0.05	15	7.4
1 month post-discontinuation of iodine-containing nutritional supplement	0.5	10	— (Free T3 not measured at this time point)
3 months post-discontinuation of iodine-containing nutritional supplement	0.5	11	3.8

The patient disclosed that she had started taking a multivitamin supplement while preparing for conception. The supplement contained selenium, iodine, iron, vitamin D, folic acid, copper, zinc, magnesium, and others. She had used the nutritional supplement for a period of five months to the time she was being reviewed at the endocrinology clinic. She was not on any other medication. She was educated and advised to discontinue the supplement with the aim that her thyroid function test would normalize. No antithyroid treatment was initiated, and no immediate evaluation with thyroid ultrasound was undertaken. A month after discontinuation, her thyroid function test revealed a serum TSH level of 0.5 mU/L and a free T4 level of 10 pmol/L. On a repeat review three months later, she remained clinically euthyroid, and her thyroid function test revealed a serum TSH of 0.5 mU/L, free T4 of 11 pmol/L, and free T3 of 3.8 pmol/L.

## Discussion

Micronutrients (iodine, selenium, iron, and vitamin D) have been shown to play important roles in regulating the thyroid gland. Iodine plays a critical role in fetal neurodevelopment and maternal metabolic regulation during pregnancy. While iodine deficiency is a well-known cause of hypothyroidism and developmental delays, excessive iodine intake, particularly through supplements, can paradoxically lead to thyrotoxicosis, a condition characterized by an overactive thyroid.

During pregnancy, thyroid physiology undergoes marked changes. Rising levels of human chorionic gonadotropin stimulate the thyroid, often lowering maternal TSH in early gestation. Estrogen increases thyroxine-binding globulin, raising total thyroid hormone levels while free hormone concentrations remain tightly regulated [5]. As such, gestational transient thyrotoxicosis (GTT) remains a differential diagnosis for the thyroid dysfunction noted in the first-trimester thyroid function test finding in this patient. GTT is, however, often associated with hyperemesis gravidarium, a finding not found in our patient. GTT is said to occur in approximately 1-3% of all pregnancies [6].

The World Health Organization (WHO) recommends a total daily intake of 250 μg of iodine for pregnant women (dietary plus supplement), typically achieved through iodized salt and supplements [7]. However, this recommendation assumes a baseline of iodine sufficiency and does not account for individual variability or underlying thyroid conditions. This patient was using a daily nutrient supplement containing iodine in preparation for pregnancy. The supplement contained the stipulated daily dose of iodine for non-pregnant individuals, but a derangement in her thyroid function test in pregnancy is relevant, bearing in mind that she had a previous thyroid condition and had been using what was deemed an innocuous supplement.

While iodine deficiency is harmful, excess iodine can also disrupt thyroid function. The Committee on Toxicity in the United Kingdom highlighted that excessive iodine intake during pregnancy may lead to thyroid hypertrophy, hyperthyroidism, and adverse maternal and fetal outcomes [8]. Acute iodine toxicity can manifest as vomiting, diarrhea, and even neurological symptoms, but chronic excess is more insidious, potentially triggering autoimmune thyroiditis or thyrotoxicosis. Excess iodine can trigger thyrotoxicosis through the Jod-Basedow phenomenon (autonomous thyroid hormone production in response to iodine load), via autoimmune activation (increased thyroid peroxidase (TPO) antibodies in susceptible individuals), or via impaired thyroid autoregulation in preexisting thyroid disease [9].

A clinical argument that can be put forward in our patient is that her deranged thyroid function test could be a mere recurrence (given a history of previous thyrotoxicosis), precipitated by her pregnancy. However, this is unlikely because her thyroid function improved to acceptable values after discontinuation of the supplement without any therapeutic intervention.

From this case, if the history of iodine-containing supplement use was not uncovered, there could have been a possibility that this patient could have been started on anti-thyroid medication unnecessarily, with its attendant side effects when the offending agent continues to be present. Not only would this have been a waste of resources but would have been a futile venture while the supplement use continued.

A WHO commentary noted that women who began iodine supplementation early in gestation had mildly decreased thyroid function compared to those with long-term iodized salt intake [7]. In women with Hashimoto’s thyroiditis, iodine supplementation was found to variably affect thyroid function, sometimes worsening hypothyroidism and, in rare cases, inducing hyperthyroidism. A study by van Heek et al. (2021) followed 20 pregnant women with Hashimoto’s thyroiditis and found that iodine supplementation altered thyroid hormone levels and antibody titers, suggesting a delicate balance of end result between thyroid deficiency and thyroid excess states [10].

A similar case was reported by Lai et al. in 2019, where a 60-year-old woman presented with thyrotoxic features after her naturopath prescribed her 12.5 mg/day of iodine complex and she was also encouraged to consume iodine through a red algae seaweed product [11]. Similarly, Mussig et al. in 2006 discussed the case of a 39-year-old German woman with a history of goiter who developed hyperthyroidism after consuming herbal tea containing kelp for four weeks [12].

A study Geller et al. over a period of nine years (2004-2013) revealed that 23,000 visits to the emergency department in the United States every year are attributed to adverse events related to dietary supplements [13]. This case report highlights the danger of iodine-containing supplement misuse in patients with either an undiagnosed or a known thyroid condition. It could bear obstetric and public health importance as a large part of intending or pregnant patients use these supplements. Secondarily, we also aim to highlight the importance of good clinical history-taking in ensuring appropriate management interventions for patients. In this case, we avoided an unnecessary prescription of anti-thyroid medications, where withdrawal of the iodine-containing supplement was all that was required to manage this patient’s deranged thyroid function test.

Attributing the thyroid dysfunction noted in this case report solely to iodine-containing nutritional supplement use could be an over-reach, but a possibility. Other sources of exogenous iodine ingestion, including a review of her full dietary intake (including the use of iodized salt or fortified foods), would have added more weight to the discussion. Moreover, measuring urinary iodine level could have equally provided stronger evidence for a causal link. The non-availability of antibody levels (thyroid-stimulating immunoglobulin, TSH receptor antibody, or TPO antibody) to exclude recurrence of her Graves’ disease at her first laboratory assessment while using the nutritional supplement is a fair consideration as a limitation of this case report, but the demonstration of reversibility to normal functions following the discontinuation of the nutritional supplement use is probably a stronger argument.

## Conclusions

A possible hypothesis from this case report suggests an awareness of the possibility of inducing or worsening thyroid dysfunction in patients with past or underlying thyroid conditions who embark on the use of iodine-containing nutritional supplements without advice. This case also reinforces the overall message regarding nutritional supplement regulation and clinical vigilance. We were also able to highlight the importance of good clinical history-taking in ensuring appropriate management interventions for patients. In this case, we avoided an unnecessary prescription of antithyroid medications where withdrawal of the iodine-containing supplement was all that was required to manage the patient’s deranged thyroid function test.
